# The role of the Wnt signalling pathway in the energy metabolism of bone remodelling

**DOI:** 10.1111/cpr.13309

**Published:** 2022-07-10

**Authors:** Mengyuan Zhu, Zhipeng Fan

**Affiliations:** ^1^ Laboratory of Molecular Signaling and Stem Cells Therapy, Beijing Key Laboratory of Tooth Regeneration and Function Reconstruction Capital Medical University School of Stomatology Beijing China; ^2^ Research Unit of Tooth Development and Regeneration Chinese Academy of Medical Sciences Beijing China

## Abstract

**Objectives:**

Bone remodelling is necessary to repair old and impaired bone caused by aging and its effects. Injury in the process of bone remodelling generally leads to the development of various bone diseases. Energy metabolism plays crucial roles in bone cell formation and function, the disorder of which will disrupt the balance between bone formation and bone resorption.

**Materials and Methods:**

Here, we review the intrinsic interactions between bone remodelling and energy metabolism and the role of the Wnt signalling pathway.

**Results:**

We found a close interplay between metabolic pathways and bone homeostasis, demonstrating that bone plays an important role in the regulation of energy balance. We also discovered that Wnt signalling is associated with multiple biological processes regulating energy metabolism in bone cells.

**Conclusions:**

Thus, targeted regulation of Wnt signalling and the recovery of the energy metabolism function of bone cells are key means for the treatment of metabolic bone diseases.

## Graphical abstract

As a critical physiological process, bone remodelling is necessary to repair impaired bone caused by daily physical load and to prevent the effects of ageing. The homeostasis of bone remodelling largely depends on the balance of energy metabolism, such as glucose, glutamine, and fatty acid metabolism, and the disturbance of which will disrupt the balance between bone formation and bone resorption. Wnt signalling pathway including critical Wnt molecules (Wnt3a, Wnt7b, Wnt10b) is significant in these life processes, which links the intrinsic interactions between bone remodelling and energy metabolism, making it a promising target for the treatment of metabolic bone diseases.

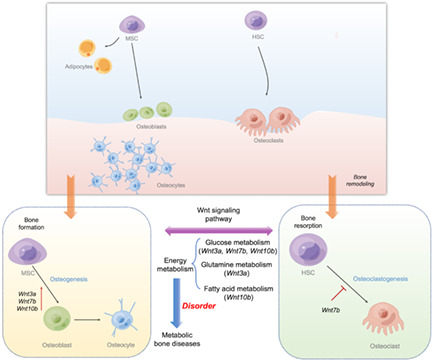

## 1 INTRODUCTION

Bone is a highly specialized and dynamic organ that regenerates constantly. The bone modelling process forms and maintains the shape of a bone, leading to the acquisition of maximum bone mass. Even after a bone is matured, the process of bone regeneration continues through periodic replacement of old bone with newly generated bone in the same place, which is known as bone remodelling. Bone remodelling is necessary to repair damaged bone due to daily physical load and to prevent the effects of ageing. The impairment of the bone remodelling process often accelerates the progression of osteoporosis, a severe health problem worldwide. The entire process of bone remodelling is a process regulated and coordinated by multiple cell types. The preservation of bone remodelling and whole‐body mineral homeostasis requires a dynamic balance between bone resorption and bone formation.^1^

Energy metabolism, like glucose, amino acid, and fatty acid metabolism, plays significant roles in the formation and function of bone cells, such as osteocytes, osteoblasts, and osteoclasts.^2^, ^3^, ^4^, ^5^, ^6^ Among the many sources of energy, glucose is the predominant source of energy and carbon for bone tissue.^7^ Previous studies using radiolabelled glucose analogues have confirmed a significant uptake of glucose by bone.^8^, ^9^ Energy metabolism disorder will disrupt the balance between bone formation and bone resorption.^4^ Recent data have identified a close interplay between metabolic pathways and bone homeostasis, demonstrating that bone plays an important role in the regulation of energy balance.^4^ In addition, recent experiments have revealed that bone is associated with energy metabolism.^10^ Several diseases share common pathophysiological features, including common regulating factors of energy expenditure and bone homeostasis, such as peroxisome proliferator‐activated receptor gamma (PPARγ), which plays a prominent role in the control of energy metabolism in bone homeostasis as well as adipose tissue.

Anorexia nervosa is a psychological disease with a characteristic of a distorted body image and self‐restricted caloric intake, and it primarily affects teenagers and young women.^11^ Patients with anorexia nervosa have less bone mass, one of the least desirable traits that threatens the health and quality of life while also decreasing whole‐body fat accumulation.^12^ Obvious signs of corporal emaciation reflect a loss of adipose tissue and lean mass because of calorie restriction, which is often associated with high energy consumption during aerobic exercise.^11^ The persistence of nutrient restriction represents an unfavourable environment that prevents cell construction and eventually damages the growth and development of bone.^13^ Under malnourished conditions, the endocrine environment is extremely disadvantageous to bone anabolism. For example, the serum levels of growth hormone are elevated, indicating a condition of hormonal resistance, as confirmed by the decrease in circulating levels of insulin‐like growth factor 1.^11^ Growth hormone resistance to promote insulin‐like growth factor 1 synthesis does not extend to the direct mediating actions of growth hormone in intermediary metabolism, such as lipolysis and protein synthesis (Table 1).^11^ However, bone remodelling markers in patients with anorexia nervosa appear to be unresponsive to the use of recombinant human growth hormone administration.

**TABLE 1:** Metabolic diseases that affect bone remodelling and homeostasis

Metabolic diseases	Vulnerable population	Symptoms of diseases	Energy metabolism pathway	Pathogenesis	References
Anorexia nervosa	Teenagers and young women	Obvious signs of corporal emaciation	Suppressed glycolysis	Suppressed bone formation and increased bone resorption	de Paula et al.^11^; Yao et al.^12^; Pando et al.^13^
Osteoporosis	Postmenopausal women and elderly	Low bone mass, degradation of the bone microstructure	Changed glutamine metabolism	Destruction of pivotal enzymes in glutamine metabolism or deterioration of mitochondria metabolism	Zhou et al.^3^; Huang et al.^14^; Singh et al.^15^
Type 2 Diabetes mellitus	Elderly people with obesity	Sudden weight loss	Impaired glucose metabolism	Affects osteoblastogenesis and disrupts osteoblastic adhesion	Li et al.^16^; Hie et al.^17^; Yang et al.^18^

**FIGURE 1:**
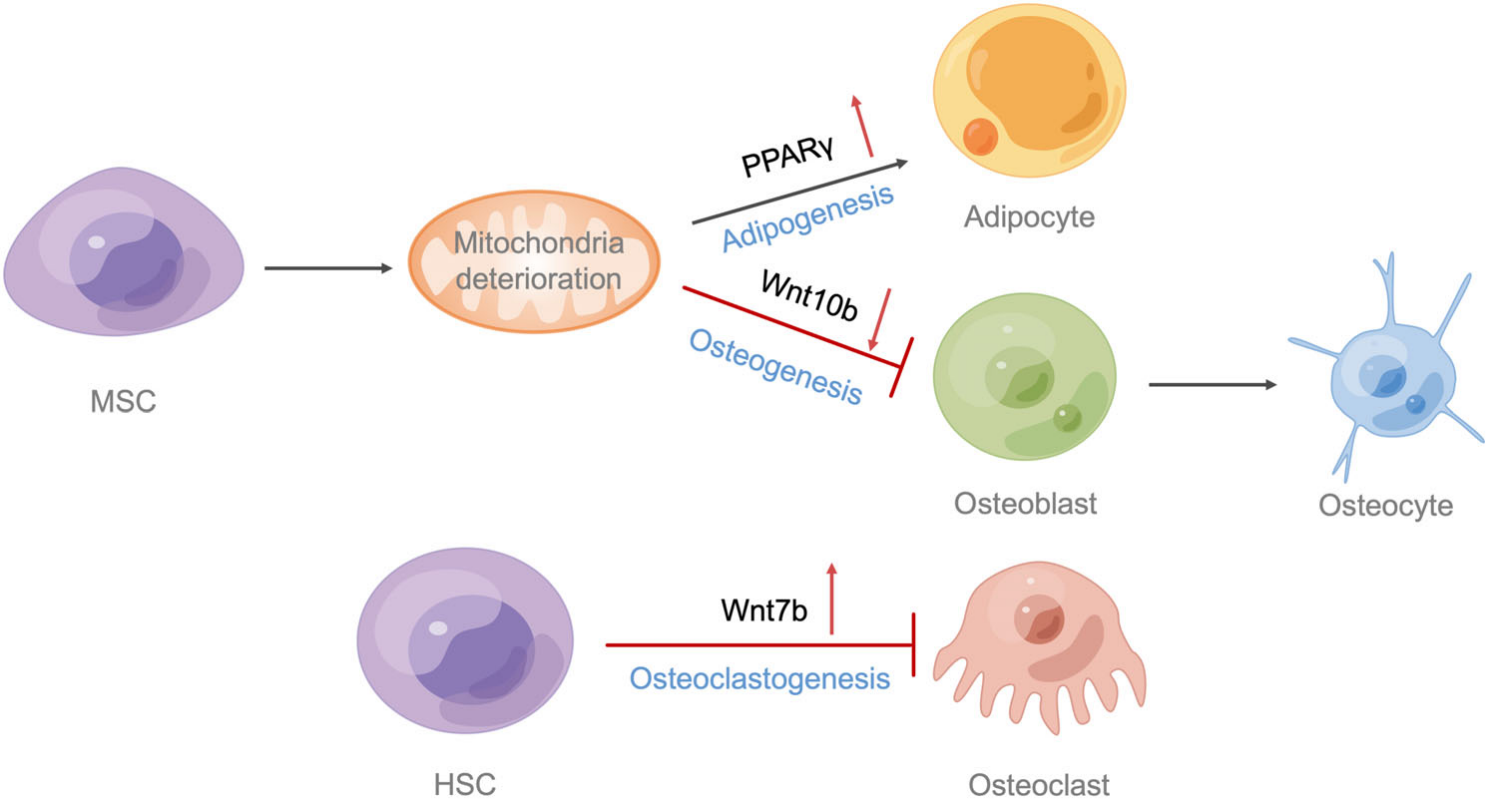
The genes of Wnt signalling pathway in bone remodelling. MSC can differentiate into adipocytes and osteoblasts, which can further differentiate into mature osteocytes. The balance between Wnt10b‐mediated osteogenesis and PPARγ‐mediated adipogenesis of MSC is disrupted when key enzymes of glutamine metabolism are impaired or mitochondrial metabolism is abnormal. Wnt7b disrupts the formation and activity of osteoclasts derived from HSC, leading to an increase in bone mass. HSC, haematopoietic stem cells; MSC, mesenchymal stem cells

Osteoporosis, being the most common metabolic bone disease, occurs mainly in postmenopausal women and elderly individuals and is characterized by low bone mass, bone microstructure degradation, and ultimately increased fracture susceptibility.^3^ The prevalence of osteoporosis in American adults is 10.3% and in women and men aged 50 years and older in China, it is 23.9% and 5.3%, respectively.^19^, ^20^ Initial findings from human genetic research reported inactivating mutations in low‐density lipoprotein receptor‐related protein 5 (Lrp5), a Wnt coreceptor, leading to osteoporosis pseudoglioma syndrome, whereas gain‐of‐function mutations result in osteosclerosis.^21^ Recently, human exome sequencing found that multiple mutations in Wnt1 are related to early stage osteoporosis and osteogenesis imperfecta.^22^, ^23^ Furthermore, Wnt16 missense mutations are related to reduced bone mineral density and incremental fracture risk in the forearm and hip.^24^ Glutamine metabolism is closely related to osteoporosis.^3^ Glutamine is critical for not only energy generation but also redox homeostasis in the homeostasis of bone, which may be a potential strategy for bone diseases like osteoporosis and osteoarthritis. Previous studies have reported that glutamine metabolism changes related to ageing in osteoporosis may disrupt the balance between osteogenesis and the adipogenesis of bone marrow mesenchymal stem cells (MSCs) through the destruction of pivotal enzymes in glutamine metabolism or the deterioration of mitochondrial metabolism (Table 1; Figure 1).^14^, ^15^ From the perspective of aetiology, early anabolic therapies combined with glutamine might be considered a treatment for osteoporosis. In fact, a glutamine precursor has been studied for application in the treatment of animal models of osteoporosis. This glutamine precursor has also been used in premature infants with glucocorticoid‐induced osteoporosis accompanied by inflammation and autoimmune diseases, and it improved growth hormone levels, increased osteocalcin concentrations, and preserved the microstructure of trabecular bone.^25^

These complex interactions may originate partially from the lineage differentiation of MSCs, and signalling pathways that influence the commitment to osteoblastogenic or adipogenic differentiation could play a crucial role in the regulation of bone and energy metabolism as well as their interactions.^4^ Wnt signalling is one of the conserved pathways involved in tissue repair, organ development, and homeostasis.^26^, ^27^, ^28^ Initiated by Wnt ligands, Wnt signalling can activate multiple intracellular signalling pathways, such as the canonical Wnt/β‐catenin signalling pathway and non‐canonical Wnt signalling pathways. Many lines of evidence indicate that Wnt signalling activation plays a significant role in the regulation of cellular bioenergetics.^12^, ^29^ Here, we focused on the role and mechanism of the Wnt signalling pathway in regulating energy metabolism in bone remodelling with the goal of providing a theoretical basis for the treatment of related metabolic bone diseases.

## 2 WNT SIGNALLING IN ENERGY METABOLISM IN BONE TISSUE

### 2.1 Energy metabolism in bone tissue

Cells take in energy from ingested or stored carbohydrates, proteins, and fats to generate adenosine 5′‐triphosphate (ATP) through a series of enzyme‐catalysed reactions. Generally, energy metabolism includes glucose metabolism, amino acid metabolism, and fatty acid metabolism.

**FIGURE 2:**
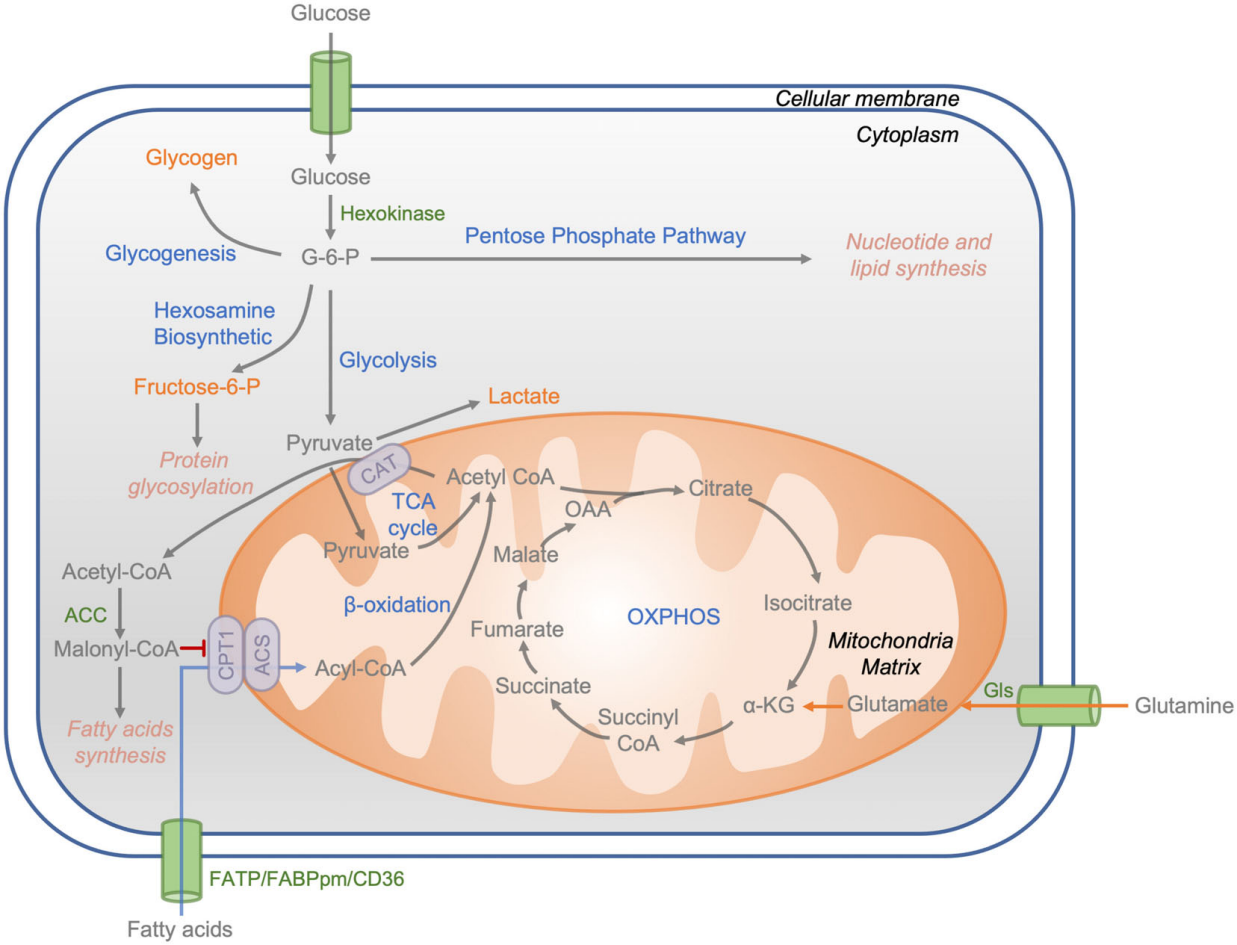
Typical energy metabolism pathways in mammalian cells. Glucose is used not only for energy production but also for intermediate metabolites in biosynthesis. Glutamine can serve as a major energy source through oxidative phosphorylation in mitochondria. This process requires the conversion of glutamine to a‐ketoglutarate. Lipid metabolism begins with the entry of fatty acids into cells. Depending on the cellular energy status, fatty acids taken up through the plasma membrane are converted into fatty acyl‐CoAs and either directly into mitochondria for β‐oxidation or stored as lipids in lipogenic tissues such as adipose tissue and liver. Major biochemical pathways are shown in blue, with the main product of each pathway denoted in orange. Several key enzymes are highlighted in green. α‐KG, a‐ketoglutarate; ACC, acetyl‐CoA carboxylase; ACS, acyl‐CoA synthetase; CAT, carnitine acyl transferase; CPT1, carnitine palmitoyl‐CoA transferase 1; FABPpm, plasma membrane‐associated fatty‐acid binding protein; FATP, fatty acid transport proteins; Fructose‐6‐P, fructose‐6‐phosphate; G‐6‐P, glucose‐6‐phosphate; Gls, glutaminase; OAA, oxaloacetate; OXPHOS, oxidative phosphorylation

First, glucose from carbohydrates is the primary source of energy and carbon for mammalian cells. Intracellular glucose is phosphorylated to glucose‐6‐phosphate by hexokinase, and glucose‐6‐phosphate can be further transformed to glycogen or then converted to generate energy and a basis for biosynthesis. For most cell types (including bone cells), most glucose‐6‐phosphate enters the key glycolysis pathway to produce pyruvate, which then enters the mitochondria to be metabolized or remains in the cytoplasm to be converted to lactate. In mitochondria, pyruvate is completely oxidized via the tricarboxylic acid (TCA) cycle (also called the Krebs cycle) and is combined with oxidative phosphorylation (OXPHOS). In addition to the key glycolysis pathway, some glycolytic intermediates, such as glucose‐6‐phosphate, could be metabolized through other mechanisms, shunted via the pentose phosphate pathway (which is important for the synthesis of nucleotides and lipids), and converted to fructose‐6‐P through the hexosamine biosynthetic pathway for protein glycosylation.^7^ Gluconeogenesis serves as an alternative source of glucose when endogenous supplies are limited (in size and rate of availability) or when the exogenous supply of carbohydrates is too low or absent.^30^ The gluconeogenesis pathway is not simply a reversal of the glycolytic pathway: the irreversible steps of glycolysis are bypassed. The process of gluconeogenesis ultimately leads to the mobilization of muscle tissue to produce glucose.^30^ The energy cost of gluconeogenesis remains a matter of debate. Although glycolysis occurs universally, gluconeogenesis is confined to the liver and kidneys and has not been reported in bone tissue; therefore, we do not discuss gluconeogenesis in detail. In general, glucose is metabolized through a variety of pathways, yet the relative proportion of each metabolic fate may depend on the respective energy and biosynthesis demand in the cell (Figure 2).

Amino acids are another source of energy for bone tissue. Glutamine is the most abundant nonessential amino acid in circulation and has multiple metabolic uses in bone cells.^31^ Glutamine metabolism is initiated by the enzyme glutaminase, which deaminates glutamine to form glutamate, an important intermediate metabolite that has many biosynthetic uses in the cell. Glutamine is transformed into α‐ketoglutarate through glutaminolysis and enters the Krebs cycle to be utilized as an alternative source of energy^31^ (Figure 2). Glutamine has been shown to be necessary for bone matrix mineralization.^32^

Lipid metabolism starts with the entry of fatty acids into bone cells. Depending on the cellular energy status, fatty acids taken up across the plasma membrane are converted into fatty acyl‐CoAs and are either directed to mitochondria for β‐oxidation or stored as lipids in lipogenic tissues such as adipose tissue and liver.^33^, ^34^ The transportation of long‐chain fatty acids (LCFA) into cells requires protein‐type transporters. Fatty acid transport proteins (FATP1‐6), plasma membrane‐associated fatty‐acid binding protein (FABPpm), and fatty acid translocase CD36 are the predominantly expressed fatty acid transporters in different types of tissues.^34^ LCFA uptake is mediated either by one or by the cooperation of multiple fatty acid transporters. Acetyl‐CoA carboxylase (ACC) is a rate‐limiting enzyme for fatty acid synthesis.^35^ Indeed, ACC carboxylates acetyl‐CoA into malonyl‐CoA, the first and regulatory step in fatty acid synthesis.^35^ Similar findings were simultaneously obtained with HMG‐CoA reductase (HMGR), the rate‐limiting enzyme of the cholesterol biosynthesis pathway.^36^ The inactivation of ACC and HMGR by their respective associated protein kinases and their reactivation by phosphatase treatment were confirmed later.^37^, ^38^ For fatty acid β‐oxidation, malonyl‐CoA is a rate‐limiting enzyme for mitochondrial fatty acid β‐oxidation and acts as an inhibitor of carnitine palmitoyl‐CoA transferase‐1 (CPT‐1).^39^ CPT‐1 catalyses the transfer of the acyl moiety of LCFA‐CoAs to carnitine and facilitates the transport of LCFA‐CoAs across the inner mitochondrial membrane^35^ (Figure 2).

### 2.2 Role of Wnt signalling in energy metabolism in bone tissue

#### 2.2.1 Wnt signalling in glucose metabolism in bone tissue

Glucose taken up by cells can be channelled catabolically into a bifurcating ATP‐generating glycolysis pathway leading to the formation of pyruvate, or shunted to the NADPH‐producing pentose phosphate pathway.^40^ Glycolysis is the main physiological way to obtain energy when the body is in a state of relative hypoxia. However, a similar phenomenon of heightened glycolysis even under aerobic conditions also occurs with cancer cells and tissues, often referred to as the Warburg effect, as glycolysis proceeds to pyruvate with an accumulation of the latter despite an aerobic condition.^40^ Aerobic glycolysis could have different pathophysiological effects in different cells; for instance, in macrophages, the apparent shift from ATP production via the TCA cycle and OXPHOS to that by glycolysis might contribute to the establishment of effective antimicrobial defences.^41^ As in cancer cells, the switch to aerobic glycolysis in neurons and glia in diseased states may be an adaptive mechanism to preserve survival under conditions of stress and energy deficiencies.^42^, ^43^ The activation of Wnt signalling promotes the expression and/or the activity of hexokinase and PFK1, important glycolytic enzymes, as well as AMP‐activated protein kinase (AMPK).^44^ The advantageous influence of the activation of Wnt signalling is partly disappears when glucose uptake is restrained.

Preclinical studies further showed that the Wnt signalling pathway participates in glucose metabolism.^45^ Mice with an Lrp5 gene null mutation develop osteopenic and glucose intolerance.^46^, ^47^ Other mouse genetic models proved that Wnt signalling plays an active role in glucose metabolism. Sclerostin knockout mice have increased glucose tolerance with insulin sensitivity and decreased white adipose reserves, suggesting an endocrine role of sclerostin, which is thought to be produced exclusively in bone (Table 2).^50^ In contrast, Lrp5 knockout mice have significantly impaired glucose tolerance and suppressed glucose‐induced insulin secretion.^47^

**TABLE 2:** The genes of the Wnt signalling pathway involved in energy metabolism in bone remodelling and bone metabolic diseases

Genes	Cell type	Metabolic processes	Biological functions	Diseases	References
Lgr4	Osteoblasts lineage cells, MC3T3‐E1 preosteoblast cells	Glycolysis	Activates canonical Wnt/β‐catenin signalling pathway	Osteoporosis	Yang et al.^18^; Carmon et al.^48^
Lgr5	MSCs	Mitochondrial fragmentation and fission	Promotes osteogenic differentiation	Osteoporosis	An et al.^49^
Lgr5	BMSCs	Glutamine metabolism and mitochondrial metabolism	Affects key enzyme in glutamine metabolism and mitochondrial metabolism	Osteoporosis	Huang et al.^14^; Singh et al.^15^
Sclerostin	Adipocytes	Glucose metabolism	Inhibits the Wnt/β‐catenin signalling pathway	Type 2 diabetes, osteoporosis and obesity	Kim et al.^50^
Wnt3a	Osteoprogenitor cells	Mitochondrial oxidative phosphorylation (OxPhos)	Acute stimulation of mitochondrial oxygen consumption		Smith et al.^51^
Wnt3a	ST2 cells	Aerobic glycolysis	Activates mTORC2 and AKT, resulting in upregulation of key glycolytic enzymes	Osteopenia	Esen et al.^29^
Wnt3a	ST2 cells	Glutamine oxidation	Stimulates energy production through increased glutamine utilization via the TCA cycle		Karner et al.^34^
Wnt7b	Bone marrow macrophages (BMMs)	Glucose metabolism	Affects glucose consumption and the expression of glucose transporters (GLUTs); impacts AKT activation during osteoclastogenesis	Osteoporosis	Wu et al.^52^
Wnt10b	MSCs	Glucose metabolism	Induces osteoblast gene expression and inhibits PPARγ2 expression	Obesity	Cawthorn et al.^53^

In addition to the role Wnt signalling plays in bone homeostasis, it is also important in glucose and lipid metabolism.^54^ In fact, a mutation in the coreceptor LRP6 of the Wnt signalling pathway was genetically linked to hyperlipidaemia, hypertension, diabetes, and osteoporosis. As a transcription factor, TCF7L2 (commonly referred to as TCF4) binds with β‐catenin of the Wnt/β‐catenin signalling pathway. Recently, owing to its non‐coding variants, the TCF4 gene has become by far the strongest susceptibility gene for Type 2 diabetes, which further proves the role that the canonical Wnt signalling pathway plays in glucose homeostasis.^54^

**FIGURE 3:**
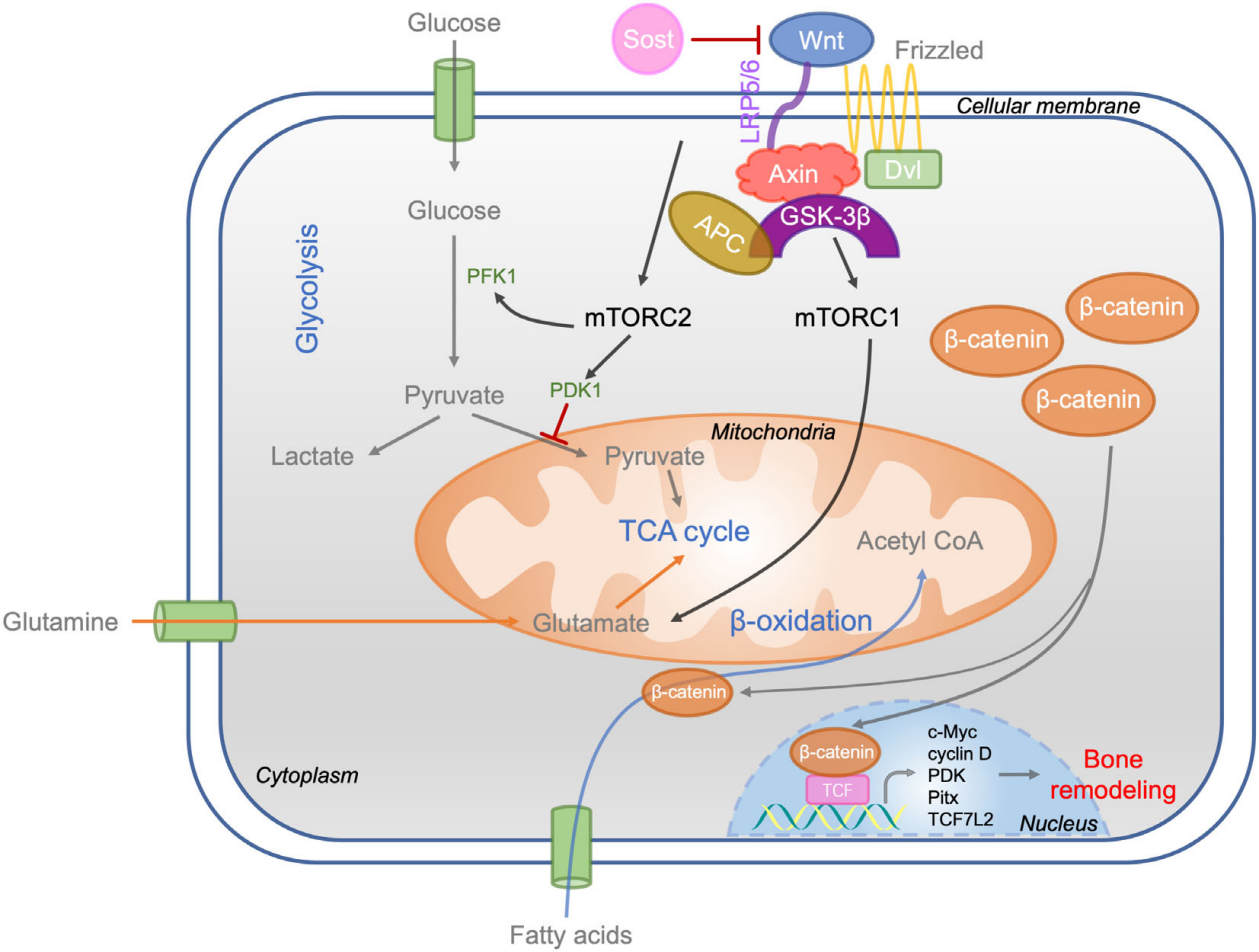
Role of Wnt signalling pathway in regulating energy metabolism. Wnt signalling promotes glycolysis by activating mTORC2 and therefore increases the expression of PFK1, a key enzyme in glycolysis. In addition, mTORC2 promotes PDK1 expression, thereby inhibiting pyruvate entry into mitochondria to participate in TCA cycle. Wnt signalling also promotes glutamine oxidation by stimulating mTORC1 and promoting the expression of glutaminase. When the content of β‐catenin changes, the catabolism of fatty acids is affected. Wnt pathway antagonists such as sclerotin inhibit the activation of Wnt signalling pathway, inhibit related metabolic processes and prevent the expression of downstream target genes related to bone remodelling. Dvl, Dishevelled; LRP5/6, low‐density lipoprotein receptor‐related protein 5/6; PDK1, pyruvate dehydrogenase kinase 1; PFK1, phosphofructokinase 1; SOST, sclerostin

TCF4 and β‐catenin are involved in the regulation of the mouse glucagon‐like peptide‐1 secretion gene, fully supporting the genetic evidence in humans.^54^ Wnt signalling together with β‐catenin are essential for islet β‐cell proliferation by Wnt ligands promoting Pitx 2, which is a direct Wnt signalling target, and cyclin D2 expression (Figure 3).^54^

#### 2.2.2 Wnt signalling in glutamine metabolism in bone tissue

Pharmacological inhibition of glutaminase can ameliorate the excessive bone formation induced by the hyperactivity of Wnt signalling in mice.^7^ In the process of osteoblast differentiation, glutamine anaplerosis partly meets the energy requirements of bone formation by responding to Wnt signalling.^21^ Wnt promotes the anaplerotic flux of glutamine into the TCA cycle by promptly increasing the level and activity of the glutaminase protein.^21^ The enzyme glutaminase catabolizes glutamine into glutamate, which is the first step of glutamine‐dependent anaplerosis. Since glutamine plays an important role in the replenishment of TCA intermediates to support energy production, it may be the main energy source activated during osteoblastogenesis. These data indicate that glutamine catabolism is an important regulatory step in achieving both the energetic and synthetic demands related to Wnt‐induced bone anabolism (Figure 3).^2^

#### 2.2.3 Wnt signalling in fatty acid metabolism in bone tissue

Growing experimental evidence suggests that regulating cellular and systemic metabolism is a primary role of Wnt signalling mediated by Lrp5.^55^ The effects of Wnt‐Lrp5 signalling on systemic metabolism may be mediated in part by actions in bone.^55^ Researchers have proposed a model in which Wnt‐Lrp5 signalling maintains insulin signalling in osteoblasts in the setting of high‐fat diet feeding and consequently affects systemic metabolism by regulating fatty acid catabolism.^55^

Sclerostin, a Wnt signalling antagonist, is produced mostly by osteocytes embedded in the bone matrix, and its ability to profoundly control bone formation has been preliminarily investigated.^56^, ^57^ After combining with low‐density lipoprotein receptor‐related protein 4 (LRP4), a sclerostin receptor, sclerostin interacts with the specific structural domain of the Wnt coreceptor LRP5/6, thus inhibiting osteoanabolic Wnt/β‐catenin signalling.^58^, ^59^, ^60^ Additionally, according to a report, Lrp5 deletion leads to a decrease in fatty acid metabolism in osteoblasts, whereas an increase is induced by the expression of the Lrp5 high‐bone‐mass variant.^61^ Previous studies have shown that sclerostin can also regulate adipose physiology.^50^, ^62^ Under each circumstance, the alteration in adipose tissue mass was related to a corresponding change in markers of Wnt/β‐catenin signalling together with the ratio of catabolism to anabolism, indicating that circulating sclerostin allows communication between bone and adipose tissue to coordinate their activity(Figure 3).^63^

Furthermore, the findings of an in vivo study suggest that sclerostin regulates not only anabolic but also catabolic metabolism, resulting in an increased fat mass and adipocyte diameter.^50^ For the in vitro study, after recombinant sclerostin treatment, the oxidation of long‐chain fatty acids was repressed, and fatty acid synthesis was enhanced, which was entirely consistent with the findings of a previous study.^50^ In addition, these data support recent findings showing that Wnt signalling could regulate metabolic ability in organs such as the liver and bone and is analogous to the phenotype of mice that lack secreted frizzled‐related protein 5, which is a secreted Wnt signalling inhibitor, generated by adipocytes.^61^, ^64^ Similar to sclerostin knockout mice, genetic ablation of secreted frizzled‐related protein 5 expression inhibits adipocyte hypertrophy and increases adipocyte metabolic capacity.^65^

## 3 WNT SIGNALLING PATHWAY AND BONE CELLS

### 3.1 Energy metabolism of bone marrow mesenchymal stem cells (BMSCs)

BMSCs, known as non‐haemopoietic multipotent mesenchymal cells, are traditionally capable of differentiating into osteoblasts, adipocytes, and chondrocytes, thereby regulating bone homeostasis.^66^, ^67^ Recently, energy metabolisms, including glucose metabolism, glutamine metabolism, and fatty acid metabolism, in BMSCs in various contexts have been consistently reported.^68^, ^69^, ^70^ Glucose is a major nutrient for BMSCs.^71^ Glutamine, as the second critical regulator after glucose, exerts an essential modulatory effect on BMSCs proliferation, lineage allocation, osteoblast specification, and even immunomodulatory properties.^3^ Fatty acids, generated from stored triacylglycerides or fat depots and released into the circulation, are degraded in the mitochondria for the generation of ATP in bone cells, while the amount that is utilized for ATP production is currently unknown.^7^

More recent evidence has shown that altered glucose uptake affects BMSCs differentiation.^9^, ^72^ Glycolysis is thought to preserve the ‘stemness’ of proliferating BMSCs.^73^ However, some recent findings highlight that aerobic glycolysis is the predominant source of energy that promotes the differentiation of BMSCs.^74^, ^75^, ^76^ Conversely, another study implied that osteogenic differentiation of BMSCs requires a metabolic switch from glycolysis to increased mitochondrial OXPHOS to ensure a sufficient energy supply.^77^ OXPHOS is not necessary for the osteogenic differentiation of human BMSCs and ST2 cells but contributes to adipogenic differentiation in human BMSCs.^29^, ^78^, ^79^

BMSCs consume and metabolize a significant amount of glutamine as they undergo differentiation into osteoblasts. As BMSCs differentiate towards osteoblasts, glutamine metabolism provides ATP through the TCA cycle.^31^ Furthermore, an integrated mechanism in a glutamine‐dependent pattern is involved in meeting energetic and synthetic demands during BMSCs differentiation.^3^ In addition to glutamine, glutaminase also promotes osteogenic differentiation of BMSCs.^80^ Wang et al.^81^ found that the glutamine metabolite α‐ketoglutarate (α‐KG) promotes the osteogenic potential of BMSCs by decreasing the accumulation of histone methylation. Taken together, these findings show that glutamine itself, glutaminase, and the glutamine metabolites α‐KG can promote the osteogenic differentiation of BMSCs.

Studies have revealed that fatty acids are second only to glucose as a main nutritional determinant for BMSCs and that fatty acid metabolism can also promote BMSCs osteogenic differentiation.^82^, ^83^ An in vitro study demonstrated that long‐chain saturated fatty acids such as palmitate can inhibit the osteogenic differentiation of BMSCs, which can be mitigated by oleate.^84^, ^85^, ^86^ Fatty acids function as an energy source to facilitate bone formation, and fatty acid deprivation suppresses osteogenic differentiation possibly by inhibiting fatty acid oxidation and the expression of specific receptors.^87^

### 3.2 Energy metabolism of osteocytes

With 90%–95% of the overall cell number in bone, osteocytes can live for more than 20 years. However, there are few data on the bioenergetics of osteocytes. Recently, an increasing number of endocrine functions have been attributed to osteocytes. By using osteocyte‐depleted mice, researchers demonstrated that osteocytes help regulate the immune system and energy metabolism.^88^ Furthermore, recent studies found that oxygen sensing downregulates bone mass in osteocytes.^89^ In addition, there is evidence that the content of glycolysis‐related proteins and mitochondria in osteocytes is highly related to oxygen‐regulated protein 150 expression.^90^, ^91^ These facts indicated that osteocytes have a high degree of glycolysis in producing energy. These results imply that osteocytes should have metabolic activity in the process of bone remodelling.

### 3.3 Energy metabolism of osteoblasts

Due to their biomass and energy consumption during the process of bone remodelling, bones are important places for nutrient absorption.^8^, ^9^, ^92^ The balance between bone formation and resorption is essential for bone homeostasis. In addition, osteoblast differentiation and its function require the integrity of nutrient utilization.^7^ Glucose has long been considered a major nutrient of osteoblasts, of which the main product is lactate. Osteoblasts consume glucose in the process of glycolysis, even the oxygen is sufficient.^93^ Aerobic glycolysis is an osteoblast metabolic characteristic as well as an indispensable function of osteoblasts. The aerobic glycolysis stimulation of preosteoblasts stabilized by HIF1α results in increased formation of bone and is alleviated through the suppression of glycolysis.^7^ Parathyroid hormone is a typical osteogenic‐promoting factor, as is Wnt signalling, and has recently been identified to promote osteoblast aerobic glycolysis.^29^, ^94^, ^95^, ^96^

It has been shown that amino acid metabolism is also significant for osteoblast differentiation.^4^ Among amino acids in circulation, glutamine is the most plentiful source of energy.^97^ There is evidence that in the TCA cycle, glutamine can be oxidized, thereby promoting energy generation in mitochondria. Besides, glutamine is a favourable replaceable fuel for osteoblasts since its metabolism promotes osteoblast capability when glucose is lacking.^2^, ^32^ Similarly, glutamine is critical in the maintenance of redox homeostasis, further improving osteoblast capability.^3^

In addition, fatty acid metabolism in osteoblasts is needed for bone acquisition depending on sex and diet.^6^ Fatty acids circulate throughout the body and exist in bone marrow sera as well.^98^ Besides, some kinds of long‐chain fatty acids can be increased and processed during osteoblast differentiation, indicating greater use of fatty acids as substrates for energy production.^7^ However, some lipids have deleterious effects on osteoblasts. Therefore, the roles of fatty acids are (1) a source of energy to promote bone formation and (2) the inhibition of deleterious effects of osteogenic differentiation, possibly through specific receptors.

### 3.4 Energy metabolism of osteoclasts

Glucose is of great importance for supporting osteoclast energy needs.^4^ Osteoclasts are reported to require a large amount of energy because of their specific performance and motility associated with bone resorption.^4^ Not only glycolysis in the cytoplasm but also the TCA cycle in mitochondria is significant for osteoclast differentiation and functions.^99^, ^100^ It was found that aerobic and anaerobic respiration both occur in the processes of osteoclast formation and function.^99^ Glycolysis is likely to function where bone resorption occurs, with ATP production and transportation, which could provide the H^+^ pump with motility and activity when bone resorption occurs.^99^ Notably, the inhibition of mitochondrial function has been proven to upregulate osteoclast functions. In summary, the formation of osteoclasts utilizes OXPHOS as a major source of bioenergy, whereas bone resorption function primarily depends on glycolysis.^4^

In recent years, the molecular mechanisms of energy metabolism regulation in the process of osteoclast formation and function have been revealed. MYC serves as an important regulator that helps metabolic reprogramming of osteoclasts to OXPHOS.^101^ Genetic research confirmed that osteoclast development in mice with osteoclast‐specific MYC deficiency was defective and therefore prevented bone loss caused by osteoporosis. Aside from MYC, HIF1α is essential in osteoclast metabolic pathway regulation. The findings indicated that hypoxia affects multiple biological processes in osteoclasts, such as glycolysis, glutamine metabolism, and ATP generation, together with the mitochondrial electron transport chain.^102^ The above effects allow rapid bone absorption in a short time and damage the cell life span. Apart from glucose, whether other substrates take part in OXPHOS during osteoclast differentiation and to what extent they contribute to the process are currently unknown.

Reactive oxygen species, which are radical forms of oxygen, occur as outcomes of mitochondrial respiration and oxidase activity. Elevated levels of reactive oxygen species have two important consequences: one is the destruction of DNA, lipids, and proteins, resulting in cell death, and the other is that they can activate specific signalling pathways.^54^ Reactive oxygen species have been proven to play significant roles in stimulating osteoclast formation and resorption.^103^, ^104^

### 3.5 Role of the Wnt signalling pathway in the energy metabolism of bone cells

Wnt/β‐catenin signalling regulates numerous osteoblast functions, including the initiation of fate determination and maturation.^105^, ^106^ β‐catenin is an important factor in the canonical Wnt signalling pathway. When the pathway is activated by any Wnt ligand, it can regulate the transcription of downstream Wnt target genes. Thus, β‐catenin is a critical target for exploring the function of this pathway.

Diabetes mellitus affects osteoblastogenesis and disrupts osteoblastic adhesion related to Wnt signalling by inhibiting AKT signalling.^16^, ^17^ By introducing PPARγ in MC‐3 T3 cells, β‐catenin, p‐AMPK, and p‐AKT signalling were activated, but p‐glycogen synthase kinase 3β (p‐GSK3β) signalling was downregulated.^107^ Meanwhile, PPARγ can significantly enhance mitochondrial biosynthesis and counteract glucose oxidation.^107^ The inhibition of GSK3β activates β‐catenin, which then promotes the differentiation of osteoblasts and MSCs.^107^ As a significant factor of energy metabolism, p‐AMPK promotes cell differentiation and is involved in insulin sensitivity through crosstalk with Wnt signalling.^108^, ^109^ In the latest research, the activation of canonical Wnt signalling ameliorated impaired glycolysis in osteoblast cells, whereas blocking Wnt/β‐catenin signalling had the opposite effect.^18^

In addition, studies have found that Wnt signalling can promote fatty acid oxidation via β‐catenin in osteoblasts.^18^ The Wnt coreceptors Lrp5/6 are important for postpartum bone recovery and osteoblast function. Recently, researchers discovered a unique function of Lrp5‐facilitating the oxidation of fatty acids in osteoblasts.^61^ Lrp5‐deficient mice have reduced bone mass after birth but increased body weight and reduced energy expenditure. In contrast, mice with a mutant Lrp5 allele of high bone mass had reduced fat content. In this case, Wnt initiates signalling downstream of Lrp5 (not Lrp6) to induce β‐catenin activation, which thereby regulates key enzyme expression in fatty acid β‐oxidation (Figure 3). Thus, Wnt‐Lrp5 signalling, apart from being associated with fundamental cellular activities related to bone fate and differentiation, participates in bone regulation and affects whole‐body energy homeostasis through pathways distinct from osteocalcin and glucose metabolism.

## 4 THE GENES IN THE WNT SIGNALLING PATHWAY INVOLVED IN THE ENERGY METABOLISM OF BONE REMODELLING

### 4.1 Lgr4

Leucine‐rich repeat‐containing G protein‐coupled receptor (GPCR) 4 (Lgr4), also known as G protein‐coupled receptor 48, is involved in many physiological and pathological reactions, such as bone remodelling and energy metabolism.^110^, ^111^, ^112^ Wnt/β‐catenin signalling is reported to activate glycolysis in a variety of tumorigenic processes.^113^, ^114^, ^115^ Once Lgr4 is deficient, the function of Wnt/β‐catenin signalling is attenuated.^116^ Lgr4 promotes osteoblast glycolysis via the Wnt/β‐catenin signalling pathway. In MC3T3‐E1 preosteoblasts, Lgr4 is required for osteoblastogenesis, and its low expression results in a decreased level of aerobic glycolysis in osteoblast cell lines (Table 2).^18^ Besides, Lgr4 is proved to mediate the amplification of Wnt/β‐catenin signalling.^48^ Lgr4 ablation harms aerobic glycolysis and osteogenic capacity, and this can be restored by activating canonical Wnt signalling in preosteoblasts. In addition, mice lacking osteoblast Lgr4 specifically (Lgr4^osb−/−^) appear to have reduced bone mass and reduced bone‐forming ability. The downregulation of osteoblast differentiation and glycolysis markers in Lgr4^osb−/−^ mice reveal the energy metabolic function of Lgr4, affording a new target for the treatment of osteoporosis.^18^ In osteoclasts, Lgr4 serves as a decoy receptor for RANKL, inhibiting osteoclast differentiation.^112^ These studies emphasize the critical role of Lgr4 during bone remodelling. Furthermore, it was found that Lgr4 is a regulator of cell metabolism, as Lgr4 ablation increases energy consumption by promoting white‐to‐brown fat conversion.^110^

### 4.2 Lgr5

Leucine‐rich repeat‐containing GPCR 5 (Lgr5), also known as G protein‐coupled receptor 49, is another target gene of Wnt, and combined with Wnt ligand R‐spondin can regulate Wnt signalling strength. In recent years, Lgr5 has been found to be a self‐renewal molecular marker in multiple organs, such as the gut, hair follicles, kidneys, and ovaries.^52^, ^117^, ^118^, ^119^, ^120^ Furthermore, Wnt signalling could be a connection between energy metabolism and bone.^45^ Canonical Wnt signalling is of great importance to the maintenance of bone homeostasis. The gain of bone mass is closely related to functional acquisition mutations in human LRP5.^121^, ^122^

Mitochondria are complex reticular organelles critical for maintaining stem cell pluripotency and differentiation capacity.^123^, ^124^, ^125^ Mitochondrial morphology is a significant regulator and indicator to judge cell function and fate.^53^, ^126^, ^127^, ^128^ A study showed that inhibiting Lgr5 expression may weaken Wnt signalling, stimulate mitochondrial fragmentation and fission in mouse bone marrow MSCs, and suppress osteogenic differentiation (Table 2).^49^ In addition, there seems to be crosstalk between ERK signalling and Wnt signalling.^129^, ^130^ The dependence of MSCs on osteoblasts requires the mediation of ERK signalling.^131^ Furthermore, studies have shown that ERK signalling promotes osteogenesis and bone growth by activating the phosphorylation level of RUNX2 and regulating transcriptional activity.^132^ Lgr5 was also found to be involved in MSCs osteogenic differentiation by regulating Wnt and ERK signalling as well as mitochondrial fusion and fission.

### 4.3 Sclerostin

Sclerostin is a Wnt inhibitor that participates in regulating bone remodelling, and it is one of the markers for bone metabolism in clinical research.^133^, ^134^, ^135^, ^136^ Specifically, sclerostin, a glycoprotein mainly secreted in the mineralized bone matrix, inhibits the Wnt/β‐catenin signalling pathway, leading to the suppression of bone formation, which is important for osteoblast development and function.^137^, ^138^ Additionally, the role of sclerostin in glucose metabolism has been proven to regulate insulin secretion and sensitivity and increase energy metabolism.^133^, ^136^, ^139^

Another research reported that sclerostin can regulate both catabolism and anabolism in adipocytes and inhibit the Wnt/β‐catenin signalling pathway, showing an endocrine function.^50^ In addition, mice lacking sclerostin have increased insulin sensitivity together with lower adipose tissue accumulation.^50^ Therefore, as a regulator of the Wnt/β‐catenin signalling pathway, sclerotin can regulate not only bone metabolism but also adipose tissue metabolism.^134^, ^140^ Since adipocytes cannot produce sclerostin, the studies suggest that sclerostin promotes communication between the bone and adipose tissue.^134^, ^140^

### 4.4 Wnt proteins

Wnt proteins are members of the secreted glycoprotein family, which regulates multiple cell functions.^141^ Wnt proteins function through canonical and non‐canonical pathway. The former depends on the stability of β‐catenin and is significant for the maintenance of bone mass. In particular, Wnt proteins integrate with Frizzled and LRP5/6 receptors, thereby stimulating downstream signalling and inhibiting the activity of GSK3β, which consequently prevents β‐catenin phosphorylation. In contrast, when lacking Wnt ligands, GSK3β phosphorylates β‐catenin, ultimately leading to its ubiquitination.^141^ Wnt pathways can be regulated by several families of secretory antagonists and regulators, such as secreted frizzled‐related proteins, which antagonize both the canonical and non‐canonical pathways as decoy receptors, and Dickkopfs and sclerostin, which can combine with LRP5/6 receptors through various mechanisms and specifically inhibit canonical Wnt signalling.^142^, ^143^

#### 4.4.1 Wnt3a

Many signalling pathways, such as Wnt3a and BMP2, can regulate the osteogenic differentiation of MSCs and stromal cells.^144^ Wnt3a and BMP2 have little effect on glycolysis but promote mitochondrial OXPHOS in osteogenic media.^51^ A dramatic increase in mitochondrial oxygen consumption in long bone and skull‐derived osteoprogenitors during osteogenic induction caused by Wnt3a or BMP2 is a mutual characteristic (Table 2).^51^

Currently, Wnt signalling is independent of β‐catenin and directly regulates glucose metabolism.^29^ In addition, Wnt3a activates downstream mammalian/mechanistic target of rapamycin complex (mTORC) 2 and AKT via LRP5 and RAC1 signalling, leading to the upregulation of key enzymes in glycolysis (Table 2).^29^ Functionally, in vitro, the regulation of metabolism is beneficial to Wnt‐promoted osteogenic differentiation; in vivo, the regulation of metabolism is related to the bone formation through LRP5 signalling.^29^

Wnt3a, Wnt7b, and Wnt10b are known to facilitate osteoblast differentiation of the ST2 cell line, and they activate glucose consumption and promote lactate production. Wnt3a could promote the expression of pyruvate dehydrogenase kinase 1, which negatively regulates pyruvate dehydrogenase activity, thereby reducing the amount of pyruvate derived from glucose entering the TCA cycle.^145^ Apart from glycolysis and fatty acid metabolism, Wnt3a also activates glutamine metabolism in the TCA cycle through mTORC1 (Table 2).^2^ In osteoblasts, glycolysis and OXPHOS can produce energy.^93^, ^95^ Osteogenic drugs such as parathyroid hormone and Wnt3a exert therapeutic effects by increasing aerobic glycolysis in osteoblasts.^29^

#### 4.4.2 Wnt7b

As a potent Wnt ligand, an in vitro study showed that Wnt7b promotes bone formation, increases bone mass, and inhibits osteoclastogenesis (Figure 1).^146^ Furthermore, increased Wnt levels in cells of the macrophage lineage markedly destroy osteoclast formation and activity, resulting in a rapid increase in bone mass.^146^ The mechanism is that Wnt7b affects the process of glucose metabolism and the activation of AKT during osteoclastogenesis (Table 2).^146^

Wnt7b is generally expressed by the osteogenic perichondrium, which benefits the development of long bones in mice, and the deletion of Wnt7b results in delayed osteogenesis in mouse embryos.^147^, ^148^ In contrast, the overexpression of Wnt7b in osteoblasts significantly promotes bone formation in mice.^149^ Genetic studies from humans and mice have provided strong evidence that Wnt signalling plays an important role in the control of bone mass. In osteoblast lineage cells, Wnt7b activates protein kinase C and mTORC1 instead of β‐catenin signalling.^147^, ^149^, ^150^ Bone anabolism by Wnt signalling is closely associated with not only glucose metabolism but also fatty acid metabolism.^21^

In vitro experiments in a study demonstrated that decreased glucose metabolism in osteoblast lineage cells is closely involved in the loss of glucose transporter 1 (Glut1), suggesting that other glucose transporters are unable to compensate for the function.^151^ Glut1 deletion might remarkably reduce ATP levels in cells and decrease the intermediate metabolites needed for energy metabolism in the process of bone formation. In addition, another in vitro study proved that Wnt7b significantly increased Glut1 protein production with little effect on mRNA levels, but the mechanism remained unclear.^151^ Wnt7b promoted Glut1 levels and increased glucose expenditure when osteoblast cell lines were cultured, and Glut1 deficiency inhibited osteoblast differentiation in vitro. As a result, part of the effect of Wnt7b in promoting bone formation is achieved by facilitating glucose metabolism in osteoblast lineage cells.

#### 4.4.3 Wnt10b

Wnt10b/β‐catenin canonical signal transduction promotes the differentiation of MSCs into osteoblast cells, while PPARγ2 promotes their differentiation into adipocytes (Figure 1).^152^, ^153^, ^154^, ^155^ Functionally, PPARγ2 and canonical Wnt signalling pathways are interconnected, since the Wnt10b/β‐catenin signalling pathway could significantly inhibit PPARγ2 activity and adipogenesis, and in comparison with this, PPARγ2 restrains the Wnt10b/β‐catenin signalling pathway as well as osteogenesis (Table 2).^152^, ^156^ Surprisingly, selectively activating the anti‐osteoblastic features of PPARγ2 suppresses Wnt10b expression, whereas selectively activating the pro‐adipocytic features of PPARγ2 has little effect on Wnt10b expression, which suggests that the two pathways could not be completely reciprocal and may be partially dependent on other mechanisms.^156^

The activities of Wnt10b in promoting osteogenic differentiation and inhibiting adipogenic differentiation have been demonstrated in numerous studies.^152^, ^153^, ^157^, ^158^ Specifically, the overexpression of Wnt10b can induce MSCs osteogenic differentiation and inhibit the expression of PPARγ2, while ectopic expression of Wnt10b in adipocytes increases animal bone mass and compensates for bone loss with ageing.^152^, ^157^ In contrast, Wnt10b‐deficient mice showed reduced bone mass and limited proliferation and differentiation ability, with an accumulation of fat in muscle cells.^153^, ^159^ Wnt10b overexpression in hyperinsulinaemia and insulin resistance models ameliorated insulin sensitivity resistance, which markedly decreased fat mass, which is beneficial to glycaemic homeostasis.

Recent studies have implicated Wnt signalling in the regulation of lipid metabolism in bone. Stimulation with the ligand Wnt10b increases fatty acid metabolism gene expression and stimulates lipid oxidation in bone. Mechanistically, the regulation by Wnt10b appears to be β‐catenin dependent as GSK3β inhibition or β‐catenin overexpression is sufficient to stimulate fatty acid metabolism in osteoblasts.^61^

## 5 CONCLUSIONS

This article reviews the role of the Wnt signalling pathway in cellular energy metabolism in bone remodelling and the related molecular mechanisms. By summarizing the new evidence, we found that the Wnt signalling pathway controls glucose metabolism, glutamine metabolism, and fatty acid metabolism in bone in connection with other signalling pathways, and the bone anabolic function of Wnt signalling is associated with increased energy metabolism in osteoblast‐lineage cells. Abnormal expression of genes related to the pathway will lead to several metabolic diseases such as osteoporosis. In addition, Wnt signalling is associated with multiple biological processes (such as osteoblast differentiation) regulating energy metabolism in bone cells, suggesting its important function in bone remodelling. Wnt molecules (Wnt3a, Wnt7b, and Wnt10b) are associated with both bone remodelling and energy metabolism, which are important bridges between these processes. However, the latest studies mostly concentrate on the impact of energy metabolism disorders on disease occurrence. Further research is still required to determine how metabolic diseases impair energy metabolism and the function of bone homeostasis regulation. Thus, targeted regulation of Wnt signalling and the recovery of the energy metabolism function of bone cells are key means for the treatment of metabolic bone diseases.

## AUTHOR CONTRIBUTIONS

Mengyuan Zhu: design and conception, search of literature, manuscript writing, creation of figure and table, and final approval of the manuscript. Zhipeng Fan: design and conception, manuscript revising, financial support, and final approval of the manuscript. All authors read and approved the final paper.

## CONFLICT OF INTEREST

The authors declare no conflict of interest.

## Data Availability

All data used to support the findings of this study are included within the article.

## References

[1] Siddiqui JA , Partridge NC . Physiological bone remodeling: systemic regulation and growth factor involvement. Physiology. 2016;31:233‐245. 27053737 10.1152/physiol.00061.2014 PMC6734079

[2] Karner CM , Esen E , Okunade AL , Patterson BW , Long F . Increased glutamine catabolism mediates bone anabolism in response to WNT signaling. J Clin Invest. 2015;125:551‐562. 25562323 10.1172/JCI78470 PMC4319407

[3] Zhou T , Yang Y , Chen Q , Xie L . Glutamine metabolism is essential for stemness of bone marrow mesenchymal stem cells and bone homeostasis. Stem Cells Int. 2019;2019:8928934. 31611919 10.1155/2019/8928934 PMC6757285

[4] Yang J , Ueharu H , Mishina Y . Energy metabolism: a newly emerging target of BMP signaling in bone homeostasis. Bone. 2020;138:115467. 32512164 10.1016/j.bone.2020.115467 PMC7423769

[5] Shum LC , White NS , Mills BN , Bentley KL , Eliseev RA . Energy metabolism in mesenchymal stem cells during osteogenic differentiation. Stem Cells Dev. 2016;25:114‐122. 26487485 10.1089/scd.2015.0193 PMC4733323

[6] Kim SP , Li Z , Zoch ML , et al. Fatty acid oxidation by the osteoblast is required for normal bone acquisition in a sex‐ and diet‐dependent manner. JCI Insight. 2017;2:e92704. 10.1172/jci.insight.92704 PMC5621897 28814665

[7] Lee W , Guntur A , Long F , Rosen C . Energy metabolism of the osteoblast: implications for osteoporosis. Endocr Rev. 2017;38:255‐266. 28472361 10.1210/er.2017-00064 PMC5460680

[8] Zoch ML , Abou DS , Clemens TL , Thorek DL , Riddle RC . In vivo radiometric analysis of glucose uptake and distribution in mouse bone. Bone Res. 2016;4:16004. 27088042 10.1038/boneres.2016.4 PMC4820746

[9] Wei J , Shimazu J , Makinistoglu MP , et al. Glucose uptake and Runx2 synergize to orchestrate osteoblast differentiation and bone formation. Cell. 2015;161:1576‐1591. 26091038 10.1016/j.cell.2015.05.029 PMC4475280

[10] DiGirolamo DJ , Clemens TL , Kousteni S . The skeleton as an endocrine organ. Nat Rev Rheumatol. 2012;8:674‐683. 23045255 10.1038/nrrheum.2012.157

[11] de Paula FJA , Rosen CJ . Structure and function of bone marrow adipocytes. Compr Physiol. 2017;8:315‐349. 29357131 10.1002/cphy.c170010

[12] Yao Q , Yu C , Zhang X , Zhang K , Guo J , Song L . Wnt/β‐catenin signaling in osteoblasts regulates global energy metabolism. Bone. 2017;97:175‐183. 28126632 10.1016/j.bone.2017.01.028

[13] Pando R , Shtaif B , Phillip M , Gat‐Yablonski G . A serum component mediates food restriction‐induced growth attenuation. Endocrinology. 2014;155:932‐940. 24456162 10.1210/en.2013-1610

[14] Huang T , Liu R , Fu X , et al. Aging reduces an ERRalpha‐directed mitochondrial glutaminase expression suppressing glutamine anaplerosis and osteogenic differentiation of mesenchymal stem cells. Stem Cells. 2017;35:411‐424. 27501743 10.1002/stem.2470

[15] Singh K , Krug L , Basu A , et al. Alpha‐ketoglutarate curbs differentiation and induces cell death in mesenchymal stromal precursors with mitochondrial dysfunction. Stem Cells. 2017;35:1704‐1718. 28398002 10.1002/stem.2629

[16] Li L , Xia Y , Wang Z , et al. Suppression of the PI3K‐Akt pathway is involved in the decreased adhesion and migration of bone marrow‐derived mesenchymal stem cells from non‐obese diabetic mice. Cell Biol Int. 2011;35:961‐966. 21449895 10.1042/CBI20100544

[17] Hie M , Iitsuka N , Otsuka T , Tsukamoto I . Insulin‐dependent diabetes mellitus decreases osteoblastogenesis associated with the inhibition of Wnt signaling through increased expression of Sost and Dkk1 and inhibition of Akt activation. Int J Mol Med. 2011;28:455‐462. 21567076 10.3892/ijmm.2011.697

[18] Yang YY , Zhou YM , Xu JZ , et al. Lgr4 promotes aerobic glycolysis and differentiation in osteoblasts via the canonical Wnt/β‐catenin pathway. J Bone Miner Res. 2021;36:1605‐1620. 33950533 10.1002/jbmr.4321

[19] Wright NC , Looker AC , Saag KG , et al. The recent prevalence of osteoporosis and low bone mass in the United States based on bone mineral density at the femoral neck or lumbar spine. J Bone Miner Res. 2014;29:2520‐2526. 24771492 10.1002/jbmr.2269 PMC4757905

[20] Zhang ZQ , Ho SC , Chen ZQ , Zhang CX , Chen YM . Reference values of bone mineral density and prevalence of osteoporosis in Chinese adults. Osteoporos Int. 2014;25:497‐507. 23800746 10.1007/s00198-013-2418-2

[21] Karner CM , Long F . Wnt signaling and cellular metabolism in osteoblasts. Cell Mol Life Sci. 2017;74:1649‐1657. 27888287 10.1007/s00018-016-2425-5 PMC5380548

[22] Fahiminiya S , Majewski J , Mort J , Moffatt P , Glorieux FH , Rauch F . Mutations in WNT1 are a cause of osteogenesis imperfecta. J Med Genet. 2013;50:345‐348. 23434763 10.1136/jmedgenet-2013-101567

[23] Laine CM , Joeng KS , Campeau PM , et al. WNT1 mutations in early‐onset osteoporosis and osteogenesis imperfecta. N Engl J Med. 2013;368:1809‐1816. 23656646 10.1056/NEJMoa1215458 PMC3709450

[24] Zheng H‐F , Tobias JH , Duncan E , et al. WNT16 influences bone mineral density, cortical bone thickness, bone strength, and osteoporotic fracture risk. PLoS Genet. 2012;8:e1002745. 22792071 10.1371/journal.pgen.1002745 PMC3390364

[25] Dobrowolski P , Tomaszewska E , Muszyński S , Blicharski T , Pierzynowski SG . Dietary 2‐oxoglutarate prevents bone loss caused by neonatal treatment with maximal dexamethasone dose. Exp Biol Med (Maywood). 2017;242:671‐682. 28178857 10.1177/1535370217693322 PMC5363691

[26] Deb A . Cell‐cell interaction in the heart via Wnt/β‐catenin pathway after cardiac injury. Cardiovasc Res. 2014;102:214‐223. 24591151 10.1093/cvr/cvu054 PMC3989450

[27] Lim X , Nusse R . Wnt signaling in skin development, homeostasis, and disease. Cold Spring Harb Perspect Biol. 2013;5:a008029. 23209129 10.1101/cshperspect.a008029 PMC3552514

[28] Zhou D , Tan R , Fu H , Liu Y . Wnt/β‐catenin signaling in kidney injury and repair: a double‐edged sword. Laboratory Investig. 2016;96:156‐167. 10.1038/labinvest.2015.153 PMC4731262 26692289

[29] Esen E , Chen J , Karner C , Okunade A , Patterson B , Long F . WNT‐LRP5 signaling induces Warburg effect through mTORC2 activation during osteoblast differentiation. Cell Metab. 2013;17:745‐755. 23623748 10.1016/j.cmet.2013.03.017 PMC3653292

[30] Schutz Y . Protein turnover, ureagenesis and gluconeogenesis. Int J Vitam Nutr Res. 2011;81:101‐107. 22139560 10.1024/0300-9831/a000064

[31] Yu Y , Newman H , Shen L , et al. Glutamine metabolism regulates proliferation and lineage allocation in skeletal stem cells. Cell Metab. 2019;29:966‐978.e964. 30773468 10.1016/j.cmet.2019.01.016 PMC7062112

[32] Brown PM , Hutchison JD , Crockett JC . Absence of glutamine supplementation prevents differentiation of murine calvarial osteoblasts to a mineralizing phenotype. Calcif Tissue Int. 2011;89:472‐482. 21972050 10.1007/s00223-011-9537-6

[33] Bickerton AST , Roberts R , Fielding BA , et al. Preferential uptake of dietary fatty acids in adipose tissue and muscle in the postprandial period. Diabetes. 2007;56:168‐176. 17192479 10.2337/db06-0822

[34] Glatz JF , Luiken JJ , Bonen A . Membrane fatty acid transporters as regulators of lipid metabolism: implications for metabolic disease. Physiol Rev. 2010;90:367‐417. 20086080 10.1152/physrev.00003.2009

[35] Angin Y , Beauloye C , Horman S , Bertrand L . Regulation of carbohydrate metabolism, lipid metabolism, and protein metabolism by AMPK. Exp Suppl. 2012;2016(107):23‐43. 10.1007/978-3-319-43589-3_2 27812975

[36] Beg ZH , Allmann DW , Gibson DM . Modulation of 3‐hydroxy‐3‐methylglutaryl coenzyme a reductase activity with cAMP and wth protein fractions of rat liver cytosol. Biochem Biophys Res Commun. 1973;54:1362‐1369. 4356818 10.1016/0006-291x(73)91137-6

[37] Beg ZH , Stonik JA , Brewer HB . Characterization and regulation of reductase kinase, a protein kinase that modulates the enzymic activity of 3‐hydroxy‐3‐methylglutaryl‐coenzyme a reductase. Proc Natl Acad Sci U S A. 1979;76:4375‐4379. 291971 10.1073/pnas.76.9.4375 PMC411577

[38] Ingebritsen TS , Parker RA , Gibson DM . Regulation of liver hydroxymethylglutaryl‐CoA reductase by a bicyclic phosphorylation system. J Biol Chem. 1981;256:1138‐1144. 6256385

[39] Bruce CR , Hoy AJ , Turner N , et al. Overexpression of carnitine palmitoyltransferase‐1 in skeletal muscle is sufficient to enhance fatty acid oxidation and improve high‐fat diet‐induced insulin resistance. Diabetes. 2009;58:550‐558. 19073774 10.2337/db08-1078 PMC2646053

[40] Tang BL . Glucose, glycolysis, and neurodegenerative diseases. J Cell Physiol. 2020;235:7653‐7662. 32239718 10.1002/jcp.29682

[41] Krejčová G , Danielová A , Nedbalová P , et al. Drosophila macrophages switch to aerobic glycolysis to mount effective antibacterial defense. Elife. 2019;8:e50414. 31609200 10.7554/eLife.50414 PMC6867711

[42] Bolaños JP , Almeida A , Moncada S . Glycolysis: a bioenergetic or a survival pathway? Trends Biochem Sci. 2010;35:145‐149. 20006513 10.1016/j.tibs.2009.10.006

[43] Vaughn AE , Deshmukh M . Glucose metabolism inhibits apoptosis in neurons and cancer cells by redox inactivation of cytochrome c. Nat Cell Biol. 2008;10:1477‐1483. 19029908 10.1038/ncb1807 PMC2626347

[44] Herzig S , Shaw R . AMPK: guardian of metabolism and mitochondrial homeostasis. Nat Rev Mol Cell Biol. 2018;19:121‐135. 28974774 10.1038/nrm.2017.95 PMC5780224

[45] Leanza G , Fontana F , Lee S‐Y , et al. Gain‐of‐function Lrp5 mutation improves bone mass and strength and delays hyperglycemia in a mouse model of insulin‐deficient diabetes. J Bone Miner Res. 2021;36:1403‐1415. 33831261 10.1002/jbmr.4303 PMC8360087

[46] Kato M , Patel MS , Levasseur R , et al. Cbfa1‐independent decrease in osteoblast proliferation, osteopenia, and persistent embryonic eye vascularization in mice deficient in Lrp5, a Wnt coreceptor. J Cell Biol. 2002;157:303‐314. 11956231 10.1083/jcb.200201089 PMC2199263

[47] Fujino T , Asaba H , Kang M‐J , et al. Low‐density lipoprotein receptor‐related protein 5 (LRP5) is essential for normal cholesterol metabolism and glucose‐induced insulin secretion. Proc Natl Acad Sci U S A. 2003;100:229‐234. 12509515 10.1073/pnas.0133792100 PMC140935

[48] Carmon KS , Gong X , Lin Q , Thomas A , Liu Q . R‐spondins function as ligands of the orphan receptors LGR4 and LGR5 to regulate Wnt/beta‐catenin signaling. Proc Natl Acad Sci U S A. 2011;108:11452‐11457. 21693646 10.1073/pnas.1106083108 PMC3136304

[49] An JH , Yang JY , Ahn BY , et al. Enhanced mitochondrial biogenesis contributes to Wnt induced osteoblastic differentiation of C3H10T1/2 cells. Bone. 2010;47:140‐150. 20399290 10.1016/j.bone.2010.04.593

[50] Kim SP , Frey JL , Li Z , et al. Sclerostin influences body composition by regulating catabolic and anabolic metabolism in adipocytes. Proc Natl Acad Sci U S A. 2017;114:E11238‐E11247. 29229807 10.1073/pnas.1707876115 PMC5748171

[51] Smith CO , Eliseev RA . Energy metabolism during osteogenic differentiation: the role of Akt. Stem Cells Dev. 2021;30:149‐162. 33307974 10.1089/scd.2020.0141 PMC7876359

[52] Barker N , Huch M , Kujala P , et al. Lgr5(+ve) stem cells drive self‐renewal in the stomach and build long‐lived gastric units in vitro. Cell Stem Cell. 2010;6:25‐36. 20085740 10.1016/j.stem.2009.11.013

[53] Karbowski M , Youle RJ . Dynamics of mitochondrial morphology in healthy cells and during apoptosis. Cell Death Differ. 2003;10:870‐880. 12867994 10.1038/sj.cdd.4401260

[54] Manolagas SC , Almeida M . Gone with the Wnts: beta‐catenin, T‐cell factor, forkhead box O, and oxidative stress in age‐dependent diseases of bone, lipid, and glucose metabolism. Mol Endocrinol. 2007;21:2605‐2614. 17622581 10.1210/me.2007-0259

[55] Kim SP , Frey JL , Li Z , Goh BC , Riddle RC . Lack of Lrp5 signaling in osteoblasts sensitizes male mice to diet‐induced disturbances in glucose metabolism. Endocrinology. 2017;158:3805‐3816. 28938444 10.1210/en.2017-00657 PMC5695825

[56] Li X , Ominsky MS , Niu Q‐T , et al. Targeted deletion of the sclerostin gene in mice results in increased bone formation and bone strength. J Bone Miner Res. 2008;23:860‐869. 18269310 10.1359/jbmr.080216

[57] van Bezooijen RL , Roelen BAJ , Visser A , et al. Sclerostin is an osteocyte‐expressed negative regulator of bone formation, but not a classical BMP antagonist. J Exp Med. 2004;199:805‐814. 15024046 10.1084/jem.20031454 PMC2212719

[58] Bourhis E , Wang W , Tam C , et al. Wnt antagonists bind through a short peptide to the first β‐propeller domain of LRP5/6. Structure. 2011;19:1433‐1442. 21944579 10.1016/j.str.2011.07.005

[59] Li X , Zhang Y , Kang H , et al. Sclerostin binds to LRP5/6 and antagonizes canonical Wnt signaling. J Biol Chem. 2005;280:19883‐19887. 15778503 10.1074/jbc.M413274200

[60] Ellies DL , Viviano B , McCarthy J , et al. Bone density ligand, sclerostin, directly interacts with LRP5 but not LRP5G171V to modulate Wnt activity. J Bone Miner Res. 2006;21:1738‐1749. 17002572 10.1359/jbmr.060810

[61] Frey JL , Li Z , Ellis JM , et al. Wnt‐Lrp5 signaling regulates fatty acid metabolism in the osteoblast. Mol Cell Biol. 2015;35:1979‐1991. 25802278 10.1128/MCB.01343-14 PMC4420919

[62] Kim SP , Da H , Li Z , et al. Lrp4 expression by adipocytes and osteoblasts differentially impacts sclerostin's endocrine effects on body composition and glucose metabolism. J Biol Chem. 2019;294:6899‐6911. 30842262 10.1074/jbc.RA118.006769 PMC6497967

[63] Kim SP , Da H , Wang L , Taketo MM , Wan M , Riddle RC . Bone‐derived sclerostin and Wnt/β‐catenin signaling regulate PDGFRα adipoprogenitor cell differentiation. FASEB J. 2021;35:e21957. 34606641 10.1096/fj.202100691R PMC8496915

[64] Boj SF , van Es JH , Huch M , et al. Diabetes risk gene and Wnt effector Tcf7l2/TCF4 controls hepatic response to perinatal and adult metabolic demand. Cell. 2012;151:1595‐1607. 23260145 10.1016/j.cell.2012.10.053

[65] Mori H , Prestwich TC , Reid MA , et al. Secreted frizzled‐related protein 5 suppresses adipocyte mitochondrial metabolism through WNT inhibition. J Clin Invest. 2012;122:2405‐2416. 22728933 10.1172/JCI63604 PMC3386832

[66] Bianco P , Robey PG . Skeletal stem cells. Development. 2015;142:1023‐1027. 25758217 10.1242/dev.102210 PMC4360182

[67] Wu J , Zhang W , Ran Q , et al. The differentiation balance of bone marrow mesenchymal stem cells is crucial to hematopoiesis. Stem Cells Int. 2018;2018:1540148. 29765406 10.1155/2018/1540148 PMC5903338

[68] Nuschke A , Rodrigues M , Wells AW , Sylakowski K , Wells A . Mesenchymal stem cells/multipotent stromal cells (MSCs) are glycolytic and thus glucose is a limiting factor of in vitro models of MSC starvation. Stem Cell Res Ther. 2016;7:179. 27906055 10.1186/s13287-016-0436-7 PMC5134064

[69] Tohyama S , Fujita J , Hishiki T , et al. Glutamine oxidation is indispensable for survival of human pluripotent stem cells. Cell Metab. 2016;23:663‐674. 27050306 10.1016/j.cmet.2016.03.001

[70] Fillmore N , Huqi A , Jaswal JS , et al. Effect of fatty acids on human bone marrow mesenchymal stem cell energy metabolism and survival. PLoS One. 2015;10:e0120257. 25768019 10.1371/journal.pone.0120257 PMC4358990

[71] Borle AB , Nichols N , Nichols G Jr . Metabolic studies of bone in vitro. I. Normal bone. J Biol Chem. 1960;235:1206‐1210. 13802861

[72] Hong M , Zhang XB , Xiang F , Fei X , Ouyang XL , Peng XC . MiR‐34a suppresses osteoblast differentiation through glycolysis inhibition by targeting lactate dehydrogenase‐a (LDHA). In Vitro Cell Dev Biol Anim. 2020;56:480‐487. 32719987 10.1007/s11626-020-00467-0

[73] van Gastel N , Carmeliet G . Metabolic regulation of skeletal cell fate and function in physiology and disease. Nat Metab. 2021;3:11‐20. 33398192 10.1038/s42255-020-00321-3

[74] Lee SY , Long F . Notch signaling suppresses glucose metabolism in mesenchymal progenitors to restrict osteoblast differentiation. J Clin Invest. 2018;128:5573‐5586. 30284985 10.1172/JCI96221 PMC6264656

[75] Jin Z , Kho J , Dawson B , et al. Nitric oxide modulates bone anabolism through regulation of osteoblast glycolysis and differentiation. J Clin Invest. 2021;131:e138935. 10.1172/JCI138935 PMC7919726 33373331

[76] Zhang L , Jiang G , Zhao X , Gong Y . Dimethyloxalylglycine promotes bone marrow mesenchymal stem cell osteogenesis via rho/ROCK signaling. Cell Physiol Biochem. 2016;39:1391‐1403. 27606625 10.1159/000447843

[77] Li Q , Gao Z , Chen Y , Guan MX . The role of mitochondria in osteogenic, adipogenic and chondrogenic differentiation of mesenchymal stem cells. Protein Cell. 2017;8:439‐445. 28271444 10.1007/s13238-017-0385-7 PMC5445026

[78] Pattappa G , Heywood HK , de Bruijn JD , Lee DA . The metabolism of human mesenchymal stem cells during proliferation and differentiation. J Cell Physiol. 2011;226:2562‐2570. 21792913 10.1002/jcp.22605

[79] Hofmann AD , Beyer M , Krause‐Buchholz U , Wobus M , Bornhäuser M , Rödel G . OXPHOS supercomplexes as a hallmark of the mitochondrial phenotype of adipogenic differentiated human MSCs. PLoS One. 2012;7:e35160. 22523573 10.1371/journal.pone.0035160 PMC3327658

[80] Skerry TM . The role of glutamate in the regulation of bone mass and architecture. J Musculoskelet Neuronal Interact. 2008;8:166‐173. 18622085

[81] Wang Y , Deng P , Liu Y , et al. Alpha‐ketoglutarate ameliorates age‐related osteoporosis via regulating histone methylations. Nat Commun. 2020;11:5596. 33154378 10.1038/s41467-020-19360-1 PMC7645772

[82] Li J , Liu X , Zuo B , Zhang L . The role of bone marrow microenvironment in governing the balance between Osteoblastogenesis and Adipogenesis. Aging Dis. 2016;7:514‐525. 27493836 10.14336/AD.2015.1206 PMC4963194

[83] van Gastel N , Stegen S , Eelen G , et al. Lipid availability determines fate of skeletal progenitor cells via SOX9. Nature. 2020;579:111‐117. 32103177 10.1038/s41586-020-2050-1 PMC7060079

[84] Schaepe K , Werner J , Glenske K , et al. ToF‐SIMS study of differentiation of human bone‐derived stromal cells: new insights into osteoporosis. Anal Bioanal Chem. 2017;409:4425‐4435. 28516281 10.1007/s00216-017-0386-7

[85] Glenske K , Schäpe K , Wieck A , et al. Effect of long term palmitate treatment on osteogenic differentiation of human mesenchymal stromal cells ‐ impact of albumin. Bone Reports. 2020;13:100707. 32913884 10.1016/j.bonr.2020.100707 PMC7472858

[86] Palomer X , Pizarro‐Delgado J , Barroso E , Vázquez‐Carrera M . Palmitic and oleic acid: the Yin and Yang of fatty acids in type 2 diabetes mellitus. Trends in Endocrinology and Metabolism: TEM. 2018;29:178‐190. 29290500 10.1016/j.tem.2017.11.009

[87] Gao B , Huang Q , Jie Q , et al. GPR120: a bi‐potential mediator to modulate the osteogenic and adipogenic differentiation of BMMSCs. Sci Rep. 2015;5:14080. 26365922 10.1038/srep14080 PMC4568495

[88] Sato M , Asada N , Kawano Y , et al. Osteocytes regulate primary lymphoid organs and fat metabolism. Cell Metab. 2013;18:749‐758. 24140021 10.1016/j.cmet.2013.09.014

[89] Spencer JA , Ferraro F , Roussakis E , et al. Direct measurement of local oxygen concentration in the bone marrow of live animals. Nature. 2014;508:269‐273. 24590072 10.1038/nature13034 PMC3984353

[90] Guo D , Keightley A , Guthrie J , Veno PA , Harris SE , Bonewald LF . Identification of osteocyte‐selective proteins. Proteomics. 2010;10:3688‐3698. 20845334 10.1002/pmic.201000306 PMC3517134

[91] Frikha‐Benayed D , Basta‐Pljakic J , Majeska RJ , Schaffler MB . Regional differences in oxidative metabolism and mitochondrial activity among cortical bone osteocytes. Bone. 2016;90:15‐22. 27260646 10.1016/j.bone.2016.05.011 PMC4970923

[92] Riddle RC , Clemens TL . Bone cell bioenergetics and skeletal energy homeostasis. Physiol Rev. 2017;97:667‐698. 28202599 10.1152/physrev.00022.2016 PMC5539406

[93] Guntur AR , Le PT , Farber CR , Rosen CJ . Bioenergetics during calvarial osteoblast differentiation reflect strain differences in bone mass. Endocrinology. 2014;155:1589‐1595. 24437492 10.1210/en.2013-1974 PMC3990840

[94] Chen J , Long F . β‐Catenin promotes bone formation and suppresses bone resorption in postnatal growing mice. J Bone Miner Res. 2013;28:1160‐1169. 23188722 10.1002/jbmr.1834 PMC3631304

[95] Esen E , Lee SY , Wice BM , Long F . PTH promotes bone anabolism by stimulating aerobic glycolysis via IGF signaling. J Bone Miner Res. 2015;30:1959‐1968. 25990470 10.1002/jbmr.2556 PMC4825329

[96] Rastogi A , Hajela A , Prakash M , et al. Teriparatide (recombinant human parathyroid hormone [1‐34]) increases foot bone remodeling in diabetic chronic Charcot neuroarthropathy: a randomized double‐blind placebo‐controlled study. J Diabetes. 2019;11:703‐710. 30632290 10.1111/1753-0407.12902

[97] Cruzat V , Macedo Rogero M , Noel Keane K , Curi R , Newsholme P . Glutamine: metabolism and immune function, supplementation and clinical translation. Nutrients. 2018;10:1564. 10.3390/nu10111564 PMC6266414 30360490

[98] Pino AM , Miranda M , Figueroa C , Rodríguez JP , Rosen CJ . Qualitative aspects of bone marrow adiposity in osteoporosis. Front Endocrinol. 2016;7:139. 10.3389/fendo.2016.00139 PMC5078474 27826285

[99] Lemma S , Sboarina M , Porporato PE , et al. Energy metabolism in osteoclast formation and activity. Int J Biochem Cell Biol. 2016;79:168‐180. 27590854 10.1016/j.biocel.2016.08.034

[100] Kim JM , Jeong D , Kang HK , Jung SY , Kang SS , Min BM . Osteoclast precursors display dynamic metabolic shifts toward accelerated glucose metabolism at an early stage of RANKL‐stimulated osteoclast differentiation. Cell Physiol Biochem. 2007;20:935‐946. 17982276 10.1159/000110454

[101] Bae S , Lee MJ , Mun SH , et al. MYC‐dependent oxidative metabolism regulates osteoclastogenesis via nuclear receptor ERRα. J Clin Invest. 2017;127:2555‐2568. 28530645 10.1172/JCI89935 PMC5490751

[102] Morten KJ , Badder L , Knowles HJ . Differential regulation of HIF‐mediated pathways increases mitochondrial metabolism and ATP production in hypoxic osteoclasts. J Pathol. 2013;229:755‐764. 23303559 10.1002/path.4159 PMC3618370

[103] Agidigbi TS , Kim C . Reactive oxygen species in osteoclast differentiation and possible pharmaceutical targets of ROS‐mediated osteoclast diseases. Int J Mol Sci. 2019;20:3576. 10.3390/ijms20143576 PMC6678498 31336616

[104] Callaway DA , Jiang JX . Reactive oxygen species and oxidative stress in osteoclastogenesis, skeletal aging and bone diseases. J Bone Miner Metab. 2015;33:359‐370. 25804315 10.1007/s00774-015-0656-4

[105] Hill TP , Später D , Taketo MM , Birchmeier W , Hartmann C . Canonical Wnt/beta‐catenin signaling prevents osteoblasts from differentiating into chondrocytes. Dev Cell. 2005;8:727‐738. 15866163 10.1016/j.devcel.2005.02.013

[106] Song L , Liu M , Ono N , Bringhurst FR , Kronenberg HM , Guo J . Loss of wnt/β‐catenin signaling causes cell fate shift of preosteoblasts from osteoblasts to adipocytes. J Bone Miner Res. 2012;27:2344‐2358. 22729939 10.1002/jbmr.1694 PMC3474875

[107] Lee Y‐H , Kim J‐S , Kim J‐E , et al. Nanoparticle mediated PPARγ gene delivery on dental implants improves osseointegration via mitochondrial biogenesis in diabetes mellitus rat model. Nanomedicine. 2017;13:1821‐1832. 28285161 10.1016/j.nano.2017.02.020

[108] Abiola M , Favier M , Christodoulou‐Vafeiadou E , Pichard AL , Martelly I , Guillet‐Deniau I . Activation of Wnt/beta‐catenin signaling increases insulin sensitivity through a reciprocal regulation of Wnt10b and SREBP‐1c in skeletal muscle cells. PloS One. 2009;4:e8509. 20041157 10.1371/journal.pone.0008509 PMC2794543

[109] Zhao J , Yue W , Zhu MJ , Sreejayan N , Du M . AMP‐activated protein kinase (AMPK) cross‐talks with canonical Wnt signaling via phosphorylation of beta‐catenin at Ser 552. Biochem Biophys Res Commun. 2010;395:146‐151. 20361929 10.1016/j.bbrc.2010.03.161 PMC2864303

[110] Wang J , Liu R , Wang F , et al. Ablation of LGR4 promotes energy expenditure by driving white‐to‐brown fat switch. Nat Cell Biol. 2013;15:1455‐1463. 24212090 10.1038/ncb2867

[111] Luo J , Zhou W , Zhou X , et al. Regulation of bone formation and remodeling by G‐protein‐coupled receptor 48. Development. 2009;136:2747‐2756. 19605502 10.1242/dev.033571 PMC2730404

[112] Luo J , Yang Z , Ma Y , et al. LGR4 is a receptor for RANKL and negatively regulates osteoclast differentiation and bone resorption. Nat Med. 2016;22:539‐546. 27064449 10.1038/nm.4076

[113] Fang Y , Shen Z‐Y , Zhan Y‐Z , et al. CD36 inhibits β‐catenin/c‐myc‐mediated glycolysis through ubiquitination of GPC4 to repress colorectal tumorigenesis. Nat Commun. 2019;10:3981. 31484922 10.1038/s41467-019-11662-3 PMC6726635

[114] Cai C‐F , Ye G‐D , Shen D‐Y , et al. Chibby suppresses aerobic glycolysis and proliferation of nasopharyngeal carcinoma via the Wnt/β‐catenin‐Lin28/let7‐PDK1 cascade. J Exp Clin Cancer Res. 2018;37:104. 29764469 10.1186/s13046-018-0769-4 PMC5952826

[115] Lecarpentier Y , Schussler O , Hébert JL , Vallée A . Multiple targets of the canonical WNT/β‐catenin signaling in cancers. Front Oncol. 2019;9:1248. 31803621 10.3389/fonc.2019.01248 PMC6876670

[116] Mancini A , Howard SR , Marelli F , et al. LGR4 deficiency results in delayed puberty through impaired Wnt/β‐catenin signaling. JCI Insight. 2020;5:e133434. 10.1172/jci.insight.133434 PMC7308048 32493844

[117] Plaks V , Brenot A , Lawson DA , et al. Lgr5‐expressing cells are sufficient and necessary for postnatal mammary gland organogenesis. Cell Rep. 2013;3:70‐78. 23352663 10.1016/j.celrep.2012.12.017 PMC3563842

[118] Barker N , Rookmaaker MB , Kujala P , et al. Lgr5(+ve) stem/progenitor cells contribute to nephron formation during kidney development. Cell Rep. 2012;2:540‐552. 22999937 10.1016/j.celrep.2012.08.018

[119] Barker N , van Es JH , Kuipers J , et al. Identification of stem cells in small intestine and colon by marker gene Lgr5. Nature. 2007;449:1003‐1007. 17934449 10.1038/nature06196

[120] Jaks V , Barker N , Kasper M , et al. Lgr5 marks cycling, yet long‐lived, hair follicle stem cells. Nat Genet. 2008;40:1291‐1299. 18849992 10.1038/ng.239

[121] Boyden LM , Mao J , Belsky J , et al. High bone density due to a mutation in LDL‐receptor‐related protein 5. N Engl J Med. 2002;346:1513‐1521. 12015390 10.1056/NEJMoa013444

[122] Little RD , Carulli JP , Del Mastro RG , et al. A mutation in the LDL receptor‐related protein 5 gene results in the autosomal dominant high‐bone‐mass trait. Am J Hum Genet. 2002;70:11‐19. 11741193 10.1086/338450 PMC419982

[123] Xu X , Duan S , Yi F , Ocampo A , Liu GH , Izpisua Belmonte JC . Mitochondrial regulation in pluripotent stem cells. Cell Metab. 2013;18:325‐332. 23850316 10.1016/j.cmet.2013.06.005

[124] Archer SL . Mitochondrial dynamics‐‐mitochondrial fission and fusion in human diseases. N Engl J Med. 2013;369:2236‐2251. 24304053 10.1056/NEJMra1215233

[125] Forni MF , Peloggia J , Trudeau K , Shirihai O , Kowaltowski AJ . Murine mesenchymal stem cell commitment to differentiation is regulated by mitochondrial dynamics. Stem Cells. 2016;34:743‐755. 26638184 10.1002/stem.2248 PMC4803524

[126] Rehman J . Empowering self‐renewal and differentiation: the role of mitochondria in stem cells. J Mol Med. 2010;88:981‐986. 20809088 10.1007/s00109-010-0678-2 PMC3006229

[127] Wanet A , Arnould T , Najimi M , Renard P . Connecting mitochondria, metabolism, and stem cell fate. Stem Cells Dev. 2015;24:1957‐1971. 26134242 10.1089/scd.2015.0117 PMC4543487

[128] Hoque A , Sivakumaran P , Bond ST , et al. Mitochondrial fission protein Drp1 inhibition promotes cardiac mesodermal differentiation of human pluripotent stem cells. Cell Death Discovery. 2018;4:39. 29531836 10.1038/s41420-018-0042-9 PMC5841367

[129] Liu Y , Xu J , Xu L , et al. Cystic fibrosis transmembrane conductance regulator mediates tenogenic differentiation of tendon‐derived stem cells and tendon repair: accelerating tendon injury healing by intervening in its downstream signaling. FASEB J. 2017;31:3800‐3815. 28495756 10.1096/fj.201601181R

[130] Červenka I , Wolf J , Mašek J , et al. Mitogen‐activated protein kinases promote WNT/beta‐catenin signaling via phosphorylation of LRP6. Mol Cell Biol. 2011;31:179‐189. 20974802 10.1128/MCB.00550-10 PMC3019858

[131] Ge C , Xiao G , Jiang D , et al. Identification and functional characterization of ERK/MAPK phosphorylation sites in the Runx2 transcription factor. J Biol Chem. 2009;284:32533‐32543. 19801668 10.1074/jbc.M109.040980 PMC2781667

[132] Lin W , Xu L , Pan Q , et al. Lgr5‐overexpressing mesenchymal stem cells augment fracture healing through regulation of Wnt/ERK signaling pathways and mitochondrial dynamics. FASEB J. 2019;33:8565‐8577. 30991839 10.1096/fj.201900082RR

[133] Daniele G , Winnier D , Mari A , et al. Sclerostin and insulin resistance in prediabetes: evidence of a cross talk between bone and glucose metabolism. Diabetes Care. 2015;38:1509‐1517. 26084344 10.2337/dc14-2989

[134] Neumann T , Hofbauer LC , Rauner M , et al. Clinical and endocrine correlates of circulating sclerostin levels in patients with type 1 diabetes mellitus. Clin Endocrinol. 2014;80:649‐655. 10.1111/cen.12364 24237244

[135] Stanik J , Kratzsch J , Landgraf K , et al. The bone markers sclerostin, osteoprotegerin, and bone‐specific alkaline phosphatase are related to insulin resistance in children and adolescents, independent of their association with growth and obesity. Horm Res Paediatr. 2019;91:1‐8. 30904905 10.1159/000497113

[136] Wędrychowicz A , Sztefko K , Starzyk JB . Sclerostin and its association with insulin resistance in children and adolescents. Bone. 2019;120:232‐238. 30055341 10.1016/j.bone.2018.07.021

[137] Weivoda MM , Youssef SJ , Oursler MJ . Sclerostin expression and functions beyond the osteocyte. Bone. 2017;96:45‐50. 27888056 10.1016/j.bone.2016.11.024 PMC5328839

[138] Redlich K , Smolen JS . Inflammatory bone loss: pathogenesis and therapeutic intervention. Nat Rev Drug Discov. 2012;11:234‐250. 22378270 10.1038/nrd3669

[139] Janssen LGM , Van Dam AD , Hanssen MJW , et al. Higher plasma Sclerostin and lower Wnt signaling gene expression in white adipose tissue of prediabetic South Asian men compared with White Caucasian men. Diabetes Metab J. 2020;44:326‐335. 31701693 10.4093/dmj.2019.0031 PMC7188965

[140] Chen D , Xie R , Shu B , et al. Wnt signaling in bone, kidney, intestine, and adipose tissue and interorgan interaction in aging. Ann N Y Acad Sci. 2019;1442:48‐60. 30101565 10.1111/nyas.13945 PMC6372353

[141] Capulli M , Paone R , Rucci N . Osteoblast and osteocyte: games without frontiers. Arch Biochem Biophys. 2014;561:3‐12. 24832390 10.1016/j.abb.2014.05.003

[142] Kawano Y , Kypta R . Secreted antagonists of the Wnt signalling pathway. J Cell Sci. 2003;116:2627‐2634. 12775774 10.1242/jcs.00623

[143] Ke HZ , Richards WG , Li X , Ominsky MS . Sclerostin and Dickkopf‐1 as therapeutic targets in bone diseases. Endocr Rev. 2012;33:747‐783. 22723594 10.1210/er.2011-1060

[144] Marie PJ . Transcription factors controlling osteoblastogenesis. Arch Biochem Biophys. 2008;473:98‐105. 18331818 10.1016/j.abb.2008.02.030

[145] Karner CM , Esen E , Chen J , Hsu F‐F , Turk J , Long F . Wnt protein signaling reduces nuclear acetyl‐CoA levels to suppress gene expression during osteoblast differentiation. J Biol Chem. 2016;291:13028‐13039. 27129247 10.1074/jbc.M115.708578 PMC4933220

[146] Wu F , Li B , Hu X , Yu F , Shi Y , Ye L . Wnt7b inhibits osteoclastogenesis AKT activation and glucose metabolic rewiring. Front Cell Dev Biol. 2021;9:771336. 34881243 10.3389/fcell.2021.771336 PMC8645835

[147] Tu X , Joeng KS , Nakayama KI , et al. Noncanonical Wnt signaling through G protein‐linked PKCdelta activation promotes bone formation. Dev Cell. 2007;12:113‐127. 17199045 10.1016/j.devcel.2006.11.00 PMC1861818

[148] Joeng KS , Long F . Wnt7b can replace Ihh to induce hypertrophic cartilage vascularization but not osteoblast differentiation during endochondral bone development. Bone Res. 2014;2:14004. 26273517 10.1038/boneres.2014.4 PMC4472126

[149] Chen J , Tu X , Esen E , et al. WNT7B promotes bone formation in part through mTORC1. PLoS Genet. 2014;10:e1004145. 24497849 10.1371/journal.pgen.1004145 PMC3907335

[150] Wu X , Tu X , Joeng KS , Hilton MJ , Williams DA , Long F . Rac1 activation controls nuclear localization of beta‐catenin during canonical Wnt signaling. Cell. 2008;133:340‐353. 18423204 10.1016/j.cell.2008.01.052 PMC2390926

[151] Chen H , Ji X , Lee W‐C , et al. Increased glycolysis mediates Wnt7b‐induced bone formation. FASEB J. 2019;33:7810‐7821. 30913395 10.1096/fj.201900201RR PMC6593878

[152] Cawthorn WP , Bree AJ , Yao Y , et al. Wnt6, Wnt10a and Wnt10b inhibit adipogenesis and stimulate osteoblastogenesis through a β‐catenin‐dependent mechanism. Bone. 2012;50:477‐489. 21872687 10.1016/j.bone.2011.08.010 PMC3261372

[153] Stevens JR , Miranda‐Carboni GA , Singer MA , Brugger SM , Lyons KM , Lane TF . Wnt10b deficiency results in age‐dependent loss of bone mass and progressive reduction of mesenchymal progenitor cells. J Bone Miner Res. 2010;25:2138‐2147. 20499361 10.1002/jbmr.118 PMC3153316

[154] Lecka‐Czernik B , Gubrij I , Moerman EJ , et al. Inhibition of Osf2/Cbfa1 expression and terminal osteoblast differentiation by PPARgamma2. J Cell Biochem. 1999;74:357‐371. 10412038

[155] Akune T , Ohba S , Kamekura S , et al. PPARgamma insufficiency enhances osteogenesis through osteoblast formation from bone marrow progenitors. J Clin Invest. 2004;113:846‐855. 15067317 10.1172/JCI19900 PMC362117

[156] Lecka‐Czernik B , Moerman EJ , Grant DF , Lehmann JM , Manolagas SC , Jilka RL . Divergent effects of selective peroxisome proliferator‐activated receptor‐gamma 2 ligands on adipocyte versus osteoblast differentiation. Endocrinology. 2002;143:2376‐2384. 12021203 10.1210/endo.143.6.8834

[157] Bennett CN , Ouyang H , Ma YL , et al. Wnt10b increases postnatal bone formation by enhancing osteoblast differentiation. J Bone Miner Res. 2007;22:1924‐1932. 17708715 10.1359/jbmr.070810

[158] Aslanidi G , Kroutov V , Philipsberg G , et al. Ectopic expression of Wnt10b decreases adiposity and improves glucose homeostasis in obese rats. Am J Physiol Endocrinol Metab. 2007;293:E726‐E736. 17578883 10.1152/ajpendo.00248.2007

[159] Vertino AM , Taylor‐Jones JM , Longo KA , et al. Wnt10b deficiency promotes coexpression of myogenic and adipogenic programs in myoblasts. Mol Biol Cell. 2005;16:2039‐2048. 15673614 10.1091/mbc.E04-08-0720 PMC1073681

